# From “Human-to-Human” to “Human-to-Non-human” – Influence Factors of Artificial Intelligence-Enabled Consumer Value Co-creation Behavior

**DOI:** 10.3389/fpsyg.2022.863313

**Published:** 2022-05-06

**Authors:** Haitao Wen, Lulu Zhang, Ao Sheng, Mingda Li, Bingfeng Guo

**Affiliations:** School of Business and Management, Jilin University, Changchun, China

**Keywords:** artificial intelligence, value co-creation, customer engagement, virtual community, S–O–R framework

## Abstract

The emergence of artificial intelligence (AI) has changed traditional methods of value co-creation. Diverging from traditional methods, this study discusses the influencing factors of AI-supported consumer value co-creation from the perspective of human-to-non-human interactions. This study adopts the stimulus–organism–response framework with consumer engagement (CE) as the intermediary to explore the impact of consumers’ personal subjective factors, community factors, and perceptions of AI technology on their value co-creating behaviors. Data were collected from 528 respondents from the Huawei Huafen Club, Xiaomi BBS, Apple China Virtual Brand, Micromobile Phone, and Lenovo communities. SPSS Amos software was used for statistical analysis, revealing that perceived personalization, autonomy, community identity, trust in AI, and self-efficacy are motivational factors that have significant effects on consumer value co-creation behaviors, in which CE plays a significant intermediary role. Our study contributes to the literature on consumer value co-creation supported by AI technology. We also offer important insights for developers of AI-enabled products and service managers.

## Introduction

With the increasing application of artificial intelligence (AI) in marketing practices and services, methods of value co-creation among economic actors are rapidly changing (Kaartemo and Helkkula, 2018; Singh et al., 2019). With traditional value co-creation, interactions between external corporate-initiated incentives and internal consumer motivations trigger participation in value co-creation activities (Palma et al., 2019; Tajvidi et al., 2021). However, the emergence of AI seems to be changing this model. AIs are designed to emulate the thinking and learning abilities of human beings, enabling machines to perceive, understand, respond, and learn (Sabherwal and Becerra-Fernandez, 2013; Bowen and Morosan, 2018). With AI, computers are no longer merely tools for repetitive tasks; they are also co-learners and co-innovators (Arthur, 2009; Zheng et al., 2017; Barile et al., 2021). As AI can gradually perform intuitive and empathic tasks (Huang and Rust, 2018), they can use their cognitive neural networks to identify consumer personalities and interact with humans to create value (Pakkala and Spohrer, 2019; Leone et al., 2021). With AI-enabled value co-creation, AI can now play the role of the value co-creation initiator (Kaartemo and Helkkula, 2018).

Several studies have investigated the impact of AI on value co-creation, value configuration, and consumer engagement (CE; Kucharska, 2019; Peltier et al., 2020). Most were written from the perspective of service providers, beneficiaries, or their resource integrators (Kaartemo and Helkkula, 2018). They paid close attention to human-to-human interactions, but they ignored human-to-non-human interactions (Kaartemo and Helkkula, 2018). Other related studies focused on AI technology and network improvements (Barile et al., 2021). Thus, the question remains of how humans and AI technologies may interact in value co-creation (Kaartemo and Helkkula, 2018; Paschen et al., 2020), as consumers are active participants (Vargo and Lusch, 2004; Payne et al., 2008; Yi and Gong, 2013).

Therefore, it is necessary to study AI’s role in value co-creation from the perspective of consumers (Xie et al., 2008). Previous studies examined consumer motivations, expectations, willingness, and associated behaviors from aspects of individual personality, environment, brand, and so on (Füller, 2010; Akman et al., 2019; Palma et al., 2019; Zhao et al., 2019). In this work, we focus on the influence of AI technology, which has already reshaped many e-commerce services (Huang and Rust, 2018; Dwivedi et al., 2021). The current research explores the technology and function of intelligent products supported by AI, which currently lacks sufficient empirical research regarding consumer attitudes toward AI (Grover et al., 2020; Balakrishnan et al., 2021). Hence, it remains difficult to determine AI’s potential impact on e-commerce. Accordingly, this study aims to answer the following two questions:

RQ1. How do consumers’ perceptions of AI technology affect their value co-creation behaviors?

RQ2. In virtual communities, how do individual and community factors influence consumers’ AI-enabled value co-creation behaviors?

The paper is structured as follows. See section “Literature Review and Theoretical Background” presents a comprehensive review of the literature. In see section “Research Model and Hypothesis Development,” research hypotheses are derived from a detailed review of the literature. The research method and academic constructs are presented in see section “Research Methodology and Data Collection.” The analysis and findings of the results are presented in see section “Results.” See section “Discussion” provides an interpretive discussion alongside a conclusion (see section “Conclusion”), implications (see section “Theoretical and Practical Implications”), limitations, and recommendations (see section “Directions for Further Studies”).

## Literature Review and Theoretical Background

### Artificial Intelligence and Value Co-creation

Value co-creation refers to the process by which product and service providers (enterprises) and beneficiaries (consumers) jointly create value via resource integration (Prahalad and Ramaswamy, 2004). According to service-dominant logic, resources, which are regarded as either operand or operant types (Vargo and Lusch, 2004; Lusch and Nambisan, 2015; Pakkala and Spohrer, 2019; Paschen et al., 2020), are at the core of value co-creation (Paschen et al., 2020), during which participants interact with each other and exchange or integrate resources to create value (Payne et al., 2008). AI technology, as an operand resource, provides technical support for consumers who participate in value co-creation and widens their participation channels (Lusch and Nambisan, 2015). However, as an operant resource, AI technology can perceive, learn, and predict consumer motivations to potentially trigger value co-creation with humans (Saviano, 2010; Akaka and Vargo, 2014; Pakkala and Spohrer, 2019).

Prior studies on AI and value co-creation fit into three focus areas: using technology to support service providers, enabling resource integration between service providers and beneficiaries, and supporting beneficiaries’ well-being (Kaartemo and Helkkula, 2018). Many researchers have sought to leverage AI to support service providers by predicting market changes, assessing the helpfulness of consumer reviews (Singh et al., 2019), and justifying complex product development decisions (Thieme et al., 2000). Notably, AI and robots can provide personalized services by understanding consumer needs and preferences, generating new interactions between humans and machines (Glushko and Nomorosa, 2013), and triggering human value creation opportunities (Kaartemo and Helkkula, 2018). Other studies have revealed the potential impact of AI technology on value co-creation and resource integration (Huang and Rust, 2018; Paschen et al., 2020), but none have closely examined the human-centered aspect of AI interaction in this domain (Ramaswamy and Ozcan, 2016; Kaartemo and Helkkula, 2018; Ostrom et al., 2019; Paschen et al., 2020).

In close proximity to the scope of this article, some scholars have recently begun to study how individuals interact with machines to jointly create value of any kind. Paschen et al. (2020), for example, found that humans and AIs play different roles as experts, creators, commanders, and reviewers in creative activities, and they bring with them different resources that guide their behaviors. Through its integration and interpretation of unstructured data (Paschen et al., 2020), AIs have been confirmed to help people make informed decisions and enhance human awareness and abilities (Rouse and Spohrer, 2018; Mele et al., 2021; Wu et al., 2021), which cyclically leads to higher levels of interaction (Sundar, 2020). Ideally, through this process, the AI system will exhibit soft skills, such as empathy (Barile et al., 2021). This process and relationship are thought to possibly enable value co-creation in the scope of the current discussion. Hence, we aim to further explore the influencing factors of consumer value co-creation on this interaction as the results likely greatly depend on the human participant’s perspectives.

### Factors Affecting Consumer Value Co-creation Supported by Artificial Intelligence

Currently, motivations for consumer value co-creation are divided into internal and external types (Palma et al., 2019), which are affected by personal, environmental, brand, societal, and other factors (Akman et al., 2019; Zhao et al., 2019), including curiosity, internal interests, and tangible rewards (Füller, 2010; Palma et al., 2019). Many of these studies were based on virtual communities in which human-to-human value co-creation behaviors were examined (Tang and Jiang, 2018; Akman et al., 2019; Zhao et al., 2019). Normally, a virtual community consists of consumers with common hobbies and brand interests (Muniz and O’guinn, 2001; Füller, 2010); hence, they are likely to participate in value co-creation. From this, we have a viable venue in which to study AI-involved co-creation activities.

In the imagined process, the roles of consumers and available resources will differ from those of traditional scenarios (Paschen et al., 2020). For example, understanding consumers’ trust in AI (TA) and their notions of AI self-efficacy (SE) are important in estimating how resource usage might change (Zhao et al., 2019; Paschen et al., 2020; Al-Kumaim et al., 2021). Notably, AI technology shows the characteristics of autonomy and personalization (Qu, 2021), but perceptions of these characteristics may be dubious for many. Based on these constraints, our research explores the influencing factors of consumer value co-creation supported by AI from consumers’ community identification (CI) and their TA, as well as their perspectives on AI personalization, autonomy, and SE.

### Stimulus–Organism–Response (S–O–R) Framework

Mehrabian proposed S–O–R in 1974. The stimulus function has a certain effect on the subject: the cognitive organism. The corresponding response can then be identified. S–O–R provides the framework of the current research, as it supplies a method for identifying and understanding the cause and effect of human behaviors in a specific environment (Tang and Jiang, 2018), while also enabling the impact of AI technical insertion on a consumer’s psychological state and the resultant behaviors to be assessed (Parboteeah et al., 2009; Animesh et al., 2011). Moreover, S–O–R provides an acceptable testing mechanism by which all of these things can be measured (Al-Kumaim et al., 2021).

## Research Model and Hypothesis Development

**FIGURE 1:**
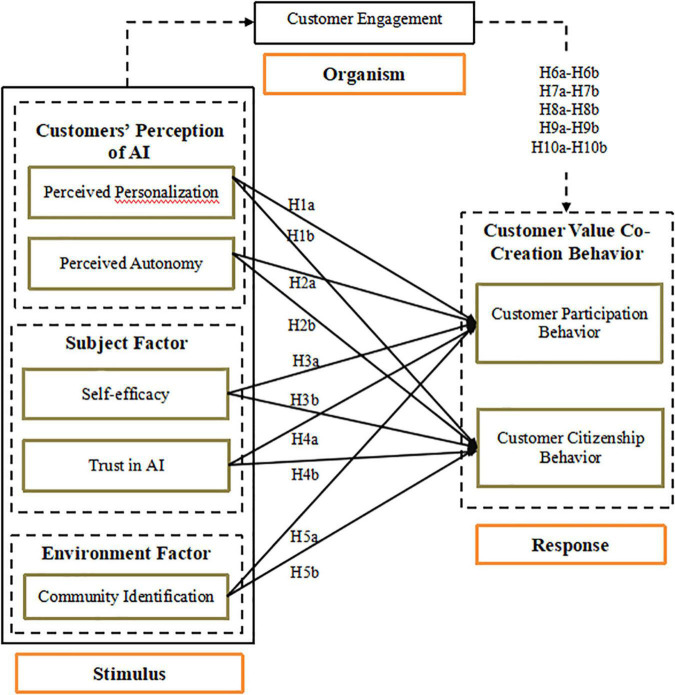
The conceptual model.

Using the S–O–R framework, this study seeks to understand the correlations among consumer perceptions of AI, subjective factors, environmental factors, CE behaviors, and consumer value co-creation behaviors (see Figure 1). S–O–R supports two major activities. First, to answer hypotheses 1 through 5 (derived below), it facilitates the examination of the positive effects of five stimulation motivations, including two consumer-perception-of-AI factors, two subjective factors, one community environmental factor, and consumer response–customer value co-creation behaviors. Second, to answer hypotheses 6 through 10 (derived below), it supports the investigation of the mediating effects of CE on the relationship among five antecedent variables and the customer behaviors affecting value co-creation (H6 to H10). All hypothesized relationships are illustrated and labeled in the framework.

### Consumer Perceptions of Artificial Intelligence and Consumer Value Co-creation Behaviors

Consumer value co-creation behavior is the dependent variable. Yi and Gong (2013) first measured this variable from two dimensions: consumer participation behavior (CPB) and consumer citizenship behavior (CCB). Follow-up research adjusted the measures according to various application scenarios (Rubio et al., 2020). In virtual communities, CPB involves seeking information and answering questions from other community members about issues, interacting among themselves and sharing best practices (Hsu et al., 2007; Revilla-Camacho et al., 2015; Yen et al., 2020). CCB reflects consumer participation in the design, development, and production of products, which may include word-of-mouth marketing and testimonies (Revilla-Camacho et al., 2015; Akman et al., 2019; Yen et al., 2020). When transplanted into an AI virtual community environment, CPB should reflect the same and similar human behaviors necessary for successful value co-creation. For example, the interface between a consumer and an AI will look the same as one between a consumer and another human; both involve proactively providing each other with information and feedback. Although CCB includes voluntary behaviors that provide value to a firm, it is not necessary for value co-creation (Groth, 2005; Bove et al., 2009; Yi et al., 2011). In the new scenario, we can imagine that if a consumer notices buggy behavior with an AI, they might be dissatisfied, but they will probably also be generally tolerant while providing authentic feedback to the AI. Furthermore, if they notice other users having difficulty using AI, they will offer help and make suggestions (Qu, 2021). In other words, CPB invokes explicitly and implicitly required social behaviors, whereas CCB encompasses voluntary or discretionary behaviors that benefit the firm (Yi and Gong, 2013). Hence, the motivation to stimulate consumers to participate in value co-creation is multifaceted.

In AI marketing research, “intelligence” is reflected in the accurate prediction of consumer demand and the provision of personalized service schemes, as well as the ability of the AI to self-learn and make decisions (Qian and Xu, 2019; Temperini et al., 2019; Zhang et al., 2019). Additionally, consumers’ perceptions of AI technology will lead to either positive or negative evaluations of AI-enabled products and services, which will, in turn, affect their interaction frequency and value perceptions (e.g., hedonic, functional, and social; Liu et al., 2017; Zhang et al., 2019; Chung et al., 2020). Hence, value co-creation should be similarly affected. This description illuminates the technical aspects of this study.

Personalization refers to how well information is tailored to a single user’s needs (Bilgihan et al., 2016). Consumer perceptions of AI providing personalized services are noticeably different from perceptions afforded to contemporary technological inputs. In this case, the technology learns consumers’ characteristics via data mining and perception techniques (Zhang et al., 2007) while accounting for consumers’ specific needs and preferences so that services can be tailored with reduced risk and lowered uncertainty (Xiao and Benbasat, 2007; Qu, 2021). For example, in online shopping platforms, AIs can infer consumer preferences based on previous browsing patterns and shopping habits. Therefore, this study assumes that most consumers are already familiar with interacting with online AIs to pursue personalized services. This fact should facilitate the measurement accuracy of the degree of consumer–AI value co-creation.

Autonomy refers to the extent to which an AI can make autonomous decisions and execute tasks correctly without requiring human feedback (Frank et al., 2021). It also refers to an AI’s ability to accommodate changes in the environment and make new decisions without intervention (Beer et al., 2014). Therefore, AIs can glean information from previous and new interactions without requiring new feedback from consumers, proactively providing humans with unexpected and perhaps better services. Moreover, Beer et al. (2014) showed that AI autonomy affects the level and frequency of user interactions. Because the products and services enabled by AI technology will seem to behave autonomously, they not only will realize the intended self-learning and autonomous improvements envisioned by AI solutions (Frank et al., 2021) but will also provide higher-quality intuitive and perhaps empathetic services to consumers, enabling humans to perceive and share some level of affection (Han and Yang, 2018; Hu et al., 2021). As implied, this will create positive perception reinforcement that cultivates harmonious relationships between humans and AIs via perceived personalization (PP) to promote value co-creation (Fiske et al., 2002). We also posit that higher AI perceived autonomy (PA) will enhance consumer value co-creation. These propositions lead to two two-part hypotheses:

H1a. In virtual AI communities, PP is positively correlated with CPB.

H1b. In virtual AI communities, PP is positively correlated with CCB.

H2a. In virtual AI communities, PA is positively correlated with CPB.

H2b. In virtual AI communities, PA is positively correlated with CCB.

### Subject Factor and Consumer Value Co-creation Behavior

Bandura (1986) defined SE as one’s belief in one’s own capacity to execute behaviors necessary to produce specific effects. A human’s perception of an AI possessing SE depends upon the human’s intuition being convinced that they are communicating with another human (or human-like) agent that can understand and respond appropriately to their needs (Li et al., 2021). Hence, when consumers sense an AI’s SE through a human–AI interaction, they will tend to accept, trust, purchase, and use AI-enabled services. Moreover, many scholars have found that the perception of an AI’s SE directly impacts consumers’ willingness to participate in AI community activities (Zhao et al., 2019). Accordingly, human participants who have higher and more mature SE are more prone to accept an AI’s provision of products and services (Al-Kumaim et al., 2021; Li et al., 2021). Therefore, this study assumes that consumers with higher SE will be likelier to participate in value co-creation activities.

Trust, a prerequisite to human-to-human interactions, has been defined by scholars as the willingness of an individual to have confidence in an entity or agent despite potential risks and losses (Cook and Wall, 1980; Chi et al., 2021). Products and services enabled by AI technology should appear to have the characteristics of anthropomorphism, autonomy, personalization and intelligence (Bartneck et al., 2009), which will enable mutual human-like social interactions, thus instilling TA (Gursoy et al., 2019; Chi et al., 2021). This level of human–AI psychological interaction will be crucial to next-generation AI-driven autonomous vehicles, and it stands to revolutionize CE via its potential to motivate resource integration in virtual communities. According to Paschen et al. (2020), the process of social resource integration clearly promotes value co-creation, and TA is key. These propositions lead to two more two-part hypotheses:

H3a. In virtual AI communities, SE is positively correlated with CPB.

H3b. In virtual AI communities, SE is positively correlated with CCB.

H4a. In virtual AI communities, TA is positively correlated with CPB.

H4b. In virtual AI communities, TA is positively correlated with CCB.

### Environmental Factors and Consumer Value Co-creation Behaviors

From a consumer perspective, CE is promoted at both the individual and group levels (Yoshida et al., 2014; Dessart et al., 2015), and CI refers to group-level camaraderie (Liao et al., 2016). In a virtual community of AI-enabled products and services, consumers will leave of their own accord if they cannot adapt or if they do not perceive value (Bateman et al., 2011; Zhou et al., 2013). Thus, CI is the glue that holds a virtual community together to support CE (Zhou et al., 2012), and it has been demonstrated that CI promotes positive interactions and value co-creation (Jian and Linghu, 2018), including the recruitment of new community members (and resources) and knowledge- and experience-sharing. Hence, we arrive at another two-part hypothesis:

H5a. In virtual AI communities, CI is positively correlated with CPB.

H5b. In virtual AI communities, CI is positively correlated with CCB.

### The Mediating Role of Consumer Engagement

To better understand the influencing factors of consumer value co-creation behaviors, previous studies used psychological variables, such as trust (Nadeem et al., 2020), satisfaction (Palma et al., 2019; Nadeem et al., 2020), and value (Zhao et al., 2019) with CE as a mediator (Yen et al., 2020). CE is driven by motivation and becomes the basis for promoting value co-creation (Jian and Linghu, 2018). Virtual communities are known to provide CE (Jian and Linghu, 2018). Therefore, CE was selected as our intermediary variable to study value co-creation behaviors. CE reflects a conglomeration of psychological states and processes, and it is enhanced via interactive consumer experiences with focal objects (e.g., AI agents; Brodie et al., 2011; Jian and Linghu, 2018). CE is stimulated by multiple antecedents (Hollebeek et al., 2019), and it traditionally represents the emotional connection between consumers and enterprises. In an AI-enabled community, if consumer needs are accurately and effectively met, CE among consumers and AI facilitators may be achieved, and the enterprise providing the AI service will benefit (Qu, 2021). According to service-dominant logic, consumers and AI agents generate value in specific situations through continued engagement (Hollebeek et al., 2019). Moreover, provided that an AI can provide human-like interactions, consumers will be just as inclined to perceive satisfaction and generate positive evaluations as a human agent, which will engender both CE and CI (Jian and Linghu, 2018).

Several studies have posited that CE mediates consumer perceptions and behavioral intentions (Yen et al., 2020; Shulga et al., 2021). Thus, given an AI’s autonomy and interactive abilities, it should be able to inspire CE (Hayes and MacLeod, 2007; Ullah et al., 2018; Prentice et al., 2020), which is known to be beneficial to consumer value co-creation. At the individual level, consumers’ self-ability and emotional attitude are inevitably related to their engagement in the community (AbdelAziz et al., 2021). At the community level, CI promotes consumers’ psychological and behavioral engagement in virtual communities (Tsai and Bagozzi, 2014), which inspires social interactions, satisfaction, and opportunities for value co-creation (Zhang et al., 2020). Based on these observations, we arrive at the final five two-part hypotheses:

H6a. CE mediates the relationship between PP and CPB.

H6b. CE mediates the relationship between PP and CCB.

H7a. CE mediates the relationship between PA and CPB.

H7b. CE mediates the relationship between PA and CCB.

H8a. CE mediates the relationship between SE and CPB.

H8b. CE mediates the relationship between SE and CCB.

H9a. CE mediates the relationship between TA and CPB.

H9b. CE mediates the association between TA and CCB.

H10a. CE mediates the relationship between CI and CPB.

H10b. CE mediates the relationship between CI and CCB.

## Research Methodology and Data Collection

### Sample and Data Collection

Community members from the Huawei Huafen Club, Xiaomi BBS, Apple China Virtual Brand, Micromobile Phone, and Lenovo communities were selected as participants. These communities were chosen for three reasons. First, they fit the commercial branding requirement and entail high CE. Huafen Club has over 40 million registered users who generate more than 200,000 posts per day (Zhao et al., 2019). Second, they fit the high-tech theme and apply service-oriented logic. For example, Xiaomi BBS provides a tripartite interaction platform that enables communication between manufacturers and promotes active consumer participation (including research and development). Third, the AI capabilities of these enterprises are mature, and their virtual communities have applied AI agents in various roles (Du et al., 2020).

We enlisted bona fide professional researchers to assist us with our survey questionnaire to ensure its suitability and relevance. The measuring scales were written in English and translated into Chinese using the progressive linguistic validation procedure to ensure the quality and accuracy of translation. We then enlisted three scholars experienced in the field and two language scholars to back-translate and cross-check the results. Several changes were then made to increase clarity, avoid misunderstandings, and reduce answering time.

We conducted a pre-survey with 90 university students familiar with Huafen Club and Xiaomi BBS, among others, who had engaged with AI-related products and services. From the pretest, 78 of 90 questionnaire responses were deemed valid. Regarding reliability and validity, the overall Cronbach’s alpha was 0.812, and that of all variables was above 0.7, indicating that the questionnaire had high internal consistency and that the results were reliable. Based on the feedback obtained from the pre-survey, we made additional adjustments for readability and comprehension.

Using the improved questionnaire, live data were collected between October 8 and 20, 2021. Target-group participants were selected using purposeful sampling techniques based on the candidates’ participation in at least one discussion of AI products and services or related activities. With the help of the official person in charge of the community, we used the settings of an online questionnaire platform, and only completed questionnaires were allowed to be submitted. After eliminating invalid responses (e.g., spurious answers, unreadable entries, and unauthorized participants), 528 of the 598 responses (88.3%) were accepted as valid. Most participants were male (71.02%), and 28.98% were female. The respondents were relatively young, aged between 18 and 30 years (85.98%). The respondent profile was determined using the frequencies and percentages shown in Supplementary Table 1.

### Measures

Responses relied on a five-point Likert scale, where 1 = “strongly disagree” and 5 = “strongly agree.” The measurement items were tailored to fit our research scope, and details are presented in Supplementary Table 2. The two variables of the AI perception dimension were PP and PA, and relevant questions were adapted from Shanahan et al. (2019) and Hu et al. (2021). For example, PP1 read “AI-enabled products and services make recommendations that match my needs,” and PA1 read “AI-enabled products and services can autonomously provide me with choices of what to do.” Next, the subject factors were SE and TA, and the questions were adapted from Chen and Chen (2021) and Delgosha and Hajiheydari (2021). For example, SE1 read, “I believe that I can use AI-enabled products and services even if there is no one around to tell me what to do as I go,” and TA1 read, “In general, I follow the advice given to me by AI-enabled products and services.” CI questions were adapted from Bagozzi and Dholakia (2006). For example, CI1 read, “I think my identity is similar to that of other community members.” CE (mediator) questions were adapted from Vivek et al. (2014). For example, CE1 read, “I would like to know more about AI-enabled products and services.” Finally, consumer value co-creation behavior included CPB and CCB, the measurement items of which were adapted from Lüthje (2004), Jang et al. (2008), Yi and Gong (2013), and Tang and Jiang (2018). For example, CPB1 read, “Through the community, I can get information about AI-enabled products and services,” and CCB1 read, “I often publish my own reviews about AI-enabled products and services to the community.”

## Results

### Reliability and Validity

To evaluate the measurement items and structure, we tested for convergent and discriminant validity. The results are shown in Supplementary Table 3. We used the loading of items and average variance extracted (AVE) for each construct to determine convergent validity (Memon et al., 2017; Hair et al., 2021). Factor loadings exceeded 0.7, and all AVE structures exceeded the threshold of 0.50 (Fornell and Larcker, 1981), indicating that the measurement model had good convergent validity. We used composite reliability to assess reliability with a threshold of > 0.7, which is considered sufficient (Memon et al., 2017; Hair et al., 2021).

Supplementary Table 4 shows that the square root of the AVE of each potential variable was greater than its correlation coefficient with other potential variables, indicating good discriminant validity (Barclay et al., 1995). The reliability coefficients for all constructs were > 0.7 and were acceptable based on the criterion of George (2011).

### Hypothesis Testing

We used IBM SPSS Amos v24 to test the path coefficients and hypotheses. The results (chi-square divided by degrees of freedom = 2.555, root-mean-square of approximation = 0.054, goodness-of-fit index = 0.874, incremental fit index = 0.917, Tucker–Lewis index = 0.907, and the comparative fit index = 0.916) indicated that the theoretical model fit well with the data.

Supplementary Table 5 shows the results of each standardized path coefficient in the model. PP has a significant impact on CPB (β = 0.168, *p* = 0.003) and CCB (β = 0.128, *p* = 0.017); thus, H1a and H1b were supported. Similarly, PA has a significant impact on CPB (β = 0.225, *p* < 0.001) and CCB (β = 0.188, *p* < 0.001); thus, H2a and H2b were supported. Meanwhile, SE, TA, and CI all have significant effects on CPB and CCB. See Supplementary Table 5 for detailed results. Therefore, H3a, H3b, H4a, H4b, H5a, and H5b were supported.

**FIGURE 2:**
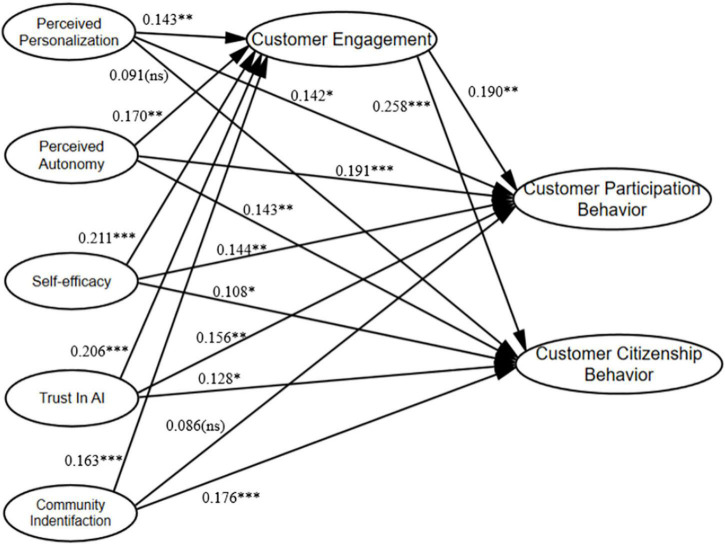
Path coefficients of the hypothesized model. *p < 0.05, ^**^p < 0.01, ^***^p < 0.001.

The mediation model shown in Figure 2 was analyzed and verified using the bootstrap method proposed by Hayes (2017). The results are shown in Supplementary Table 6. The upper and lower limits of the 95% confidence intervals were met, and the 95% CI did not contain zero, indicating that the mediating effects of CE on the relationship between PP and consumer value co-creation behaviors are significant. Thus, H6a, H6b, H7a, H7b, H8a, H8b, H9a, H9b, H10a, and H10b were all supported. CE therefore plays a complete intermediary role between PP and CCB, as well as between CI and CCB, while it plays a partial intermediary role in the relationship between other variables.

## Discussion

In virtual AI communities, we confirmed that perceived human–AI interactions consisting of PP and PA have a significant correlation to consumer value co-creation behaviors owing to the support of hypotheses H1a, H2a, H1b, and H2b. Therefore, deductively, PP is the key driver promoting consumer value co-creation behaviors in virtual AI communities, and AI-enabled products and services associated with this ability will be very sensitive to consumer needs (Shanahan et al., 2019; Qu, 2021). These findings demonstrate that AI-driven products and services can adapt to community scenarios to reassure consumers that they are understood and are being taken seriously. Simultaneously, in virtual AI communities, PP facilitates a positive consumer interactive experience (Chen et al., 2021), which is likely to inspire them to recommend the experience to their friends. Therefore, the path to value co-creation is clear. We found that AI with high levels of autonomy will excel at independently observing and analyzing the needs of consumers while offering suggestions (Hu et al., 2021). PA has the strongest relationship with CPB in virtual AI communities. Thus, when consumers’ needs are accurately and effectively met, they are likely to positively evaluate the AI technology and the enterprise providing it, further encouraging them to participate in enterprise activities (Qu, 2021). The nearly imperceptible cyclic psychological reinforcement provided by this phenomenon is expected to greatly facilitate value co-creation in virtual AI communities.

Importantly, in virtual AI communities, SE and TA were found to be positively correlated with consumer value co-creation behaviors, as hypotheses H3a, H4a, H3b, and H4b were supported. Our results show that AI SE and consumer TA positively reinforce value co-creation in virtual AI communities. It is important for consumers to have confidence when using AI products and services so that high AI SE can be perceived. Consumers who do so are more likely than those who do not to accept and engage with the AI agent, which will behaviorally and cognitively increase value co-creation intentions in virtual AI communities (AbdelAziz et al., 2021; Li et al., 2021). Thus, SE is a key variable for providers to consider when encouraging consumers to participate in human–AI value co-creation. In addition to the already verified relationship between humans and communities, we have now shown that a consumer’s TA will encourage human–AI value co-creation, and trust is the main facilitator.

We also identified that in virtual AI communities, CI is positively related to consumer co-creation behaviors, and it has the strongest relationship to CCB, because hypotheses H5a and H5b were supported. The results show that under the influence of CI, consumers in virtual AI communities will better integrate into the community and accept the value co-creation behaviors advocated by the AI agent, thus forming loyalty (Chen, 2021). In turn, customers with high CI are more willing to communicate with community members and increase their value co-creation behaviors (Liu et al., 2015). Therefore, consumers with high CI will consider themselves inseparable members of the virtual AI community and will seek more opportunities to participate in value co-creation.

Our study also found that in virtual AI communities, CE mediates the association between the antecedent variables of consumer value co-creation. First, we confirmed that PA and PP will improve consumer satisfaction, further increasing CE (Shanahan et al., 2019; Prentice et al., 2020). Human–AI interactions in this environment will enhance positive consumer experiences (Chen et al., 2021). Notably, the subject factor was also found to influence CE, which will further motivate consumers with high SE and TA to use AI-enabled products and services (Liu et al., 2017; Bravo et al., 2020; Chi et al., 2021). Moreover, CI is the best way to stimulate CE (Tsai and Bagozzi, 2014), which again strengthens consumer loyalty to AI-enabled products and services. According to the CE service system model proposed by Jaakkola and Alexander (2014), CE is the basis of value co-creation (Kumar and Pansari, 2016; Storbacka et al., 2016). It also facilitates resource integration service provision in virtual AI communities (Jian and Linghu, 2018). Through the intermediary function of CE, consumers will therefore maintain a positive evaluation of enterprise AI technologies, which, of course, further promotes value co-creation.

Our study focused on consumers predisposed to AI services. In consideration of those who do not, we expect that their SE may not immediately recognize an AI’s SE. Hence, they may lack confidence and trust, or they may even fear the AI agent. However, perhaps by utilizing immersive tutorials, interactive instructions, or similar methods, these consumers will more quickly transition to fit the demographic studied. Such tools ought to be deployed with marketing enticements (e.g., coupons, credits, or game tokens). The opportunities are vast.

## Conclusion

Contemporary value co-creation activities are well understood in terms of human-to-human and human-to-community relationships. However, AI-enabled products and services differ from contemporary scenarios in that AI agents possess background intelligent capabilities tantamount to human agents, which may more powerfully and intuitively comprehend consumer personalities, preferences, and motivations that may drive human–AI value co-creation. To determine the relevant correlations among the relevant variables of this new construct, we drew on previous literature and related theories and leveraged the S–O–R framework to investigate the value co-creation potential of consumers in virtual AI communities.

Consumer value co-creation behavior is the dependent variable, which is determined from CPB and CCB. Noting that consumers’ perceptions of AI were influencing factors, both humans and AI agents were assumed to possess PP, PA, SE, TA, and CI. Notably, those five variables have independent and supportive relationships with CPB and CCB. Hence, to fully understand consumer value co-creation behaviors in an AI-driven virtual community, the myriad relationships among the seven independent variables were determined. In summary, by applying statistical analysis to questionnaire results and rationalizing the perspectives of human consumers in virtual AI communities, PP, PA, SE, TA, and CI were all found to positively correlate with CPB and CCB, respectively (see H1–H10).

## Theoretical and Practical Implications

Our results provide both consumers and developers of AI-enabled products and services with new insights into the potential of AI-driven value co-creation. AI agents, like humans, can analyze the current situation, understand how consumers select and use products and services, and contribute to the research and development of product improvements. The domain of value co-creation can now be extended to AI virtual communities. Few scholars have touched upon this issue, but we now know that the differences between AI-enabled products and services are overcome by the same intrinsic variables used in contemporary theory. Therefore, the question can now move beyond “whether” value co-creation can take place in AI virtual communities to “how” and “how much better.” The major implication is that the power of AI and its associative neural network architectures can not only emulate human agents and support virtual communities but can also achieve superhuman performance as a facilitator, a marketeer, a recruiter, and a moderator.

Noting AI agents’ superior predictive power, faster neural processes, and near-infinite recollection, advancements in virtual AI communities must be approached with care and caution. Corporations must attend to consumer needs first, and the resources used in value co-creation should be considered human-driven. To maintain a productive and positive value co-creation environment, developers must be considerate (ethically, regulatorily, and statutorily) of human privacy concerns while enabling consumers to divulge only the information they wish to share. Simultaneously, consumers must be treated as protected agents; hence, overt manipulation and pressurized tactics must be avoided at all costs. Instead, consumers must be well-trained, respected, and empowered to comprehensively understand how the community operates and that AI agents are trustworthy by their own accord. Furthermore, human community needs must remain paramount.

Finally, for enterprises that manufacture AI-enabled products and services, this study provides several actionable methods for enhancing consumer CI and increasing opportunities for value co-creation between enterprises and consumers.

## Directions for Further Studies

Although this study makes a significant contribution to the construct of virtual AI community-based value co-creation, it has some limitations that should be considered.

First, consumer perceptions of AI technology must be further studied alongside the application of AI-driven virtual communities, as the extant literature on the topic is immature. In this study, we used the best guidance available and made relevant assumptions. Furthermore, the contributions of AI agents to value co-creation may introduce new and unexpected factors; we selected the best-known variables from theory while evaluating them from a consumer perspective.

Second, we facilitated the dependability of our results by choosing knowledgeable participants from mature brand-name virtual communities. The responses and behaviors of non-AI-savvy people will also need to be studied. With potential new developments in AI-driven virtual communities, there may soon be new types that apply divergent business approaches and socio-cultural rules. This will require a longitudinal examination. Relatedly, no virtual communities yet exist that explicitly provide AI-enabled products and services in support of value co-creation. We only determined that it is possible, and we provided the basic elements of a roadmap to those ends.

Third, we did not account for regional and cultural differences in our participant selection. This study was conducted in China, and the data collected reflected aspects of the government, the economy, and the culture, which largely ignores differences between business types, provinces, and communities. With further improvements in AI technology, researchers should consider conducting more generalizable research on a global scale. Alternatively, it would be interesting to learn more about how AI agents adapt to the governments, economies, and cultures into which they are placed.

## Data Availability Statement

The raw data supporting the conclusions of this article will be made available by the authors, without undue reservation.

## Author Contributions

HW and LZ designed the work, analyzed the data, and drafted the work. AS and BG worked on the results and revised the work. ML analyzed and interpreted the data. All authors have read and agreed to the published version of the manuscript.

## Conflict of Interest

The authors declare that the research was conducted in the absence of any commercial or financial relationships that could be construed as a potential conflict of interest.

## Publisher’s Note

All claims expressed in this article are solely those of the authors and do not necessarily represent those of their affiliated organizations, or those of the publisher, the editors and the reviewers. Any product that may be evaluated in this article, or claim that may be made by its manufacturer, is not guaranteed or endorsed by the publisher.
