# Supplementary material for: From “Human-to-Human” to “Human-to-Non-human” – Influence Factors of Artificial Intelligence-Enabled Consumer Value Co-creation Behavior
Source: Front Psychol. 2022 May 6;13:863313. doi: 10.3389/fpsyg.2022.863313 (PMC9120962; doi:10.3389/fpsyg.2022.863313)
Supplement: Supplementary file 1 [file Table_1.DOCX]

Supplementary Material

# Supplementary Figures and Tables

Here is the relevant supplementary materials for the above mentioned study.

## Supplementary Tables

**Table 1.** Respondent Demographic Profiles

| Respondent Information | Data category | Frequency  （N=528） | Percentage  （100%） |
| --- | --- | --- | --- |
| Gender | Male | 375 | 71.02 |
|  | Female | 153 | 28.98 |
| Age | Below 18 | 17 | 3.22 |
|  | 18-24 | 194 | 36.74 |
|  | 25-30 | 260 | 49.24 |
|  | 30-40 | 49 | 9.28 |
|  | 40-50 | 7 | 1.33 |
|  | Above 50 | 1 | 0.19 |
| Level of Education | Below bachelor’s degree | 29 | 5.49 |
|  | College degree | 225 | 42.61 |
|  | Bachelor’s degree | 255 | 48.30 |
|  | Master’s degree or above | 19 | 3.60 |
| Profession | National officials | 33 | 6.25 |
|  | Students | 75 | 14.20 |
|  | Education industry | 101 | 19.13 |
|  | Service industry | 116 | 21.97 |
|  | Finance | 60 | 11.36 |
|  | Engineer | 27 | 5.11 |
|  | Computer industry | 27 | 5.11 |
|  | Sales industry | 56 | 10.61 |
|  | Freelance | 28 | 5.30 |
|  | Retired or unemployed | 4 | 0.76 |
|  | Others | 1 | 0.19 |

**Table 2.** Items related to Customer Value Co-Creation factors

| **Factor** | **Item Code** | **Adopted items** | **Source of Items** |
| --- | --- | --- | --- |
| **Perceived Personalization** | PP1 | AI-enabled products and service makes recommendations that match my needs. | Shanahan et al., 2019; Srinivasan et al., 2002 |
|  | PP2 | I think that AI-enabled products and service enables me to order products that are tailor-made for me. |  |
|  | PP3 | Overall, AI-enabled products and service is tailored-made to my situation. |  |
|  | PP4 | AI-enabled products and service makes me feel that I am a unique customer. |  |
|  | PP5 | I believe that AI-enabled products and service is customized to my needs. |  |
| **Perceived Autonomy** | PA1 | AI-enabled products and service can autonomously provide me choices of what to do. | Qian Hu et al., 2021 |
|  | PA2 | AI-enabled products and service can independently provide me recommendations for action plans for assigned matters. |  |
|  | PA3 | AI-enabled products and service can independently recommend an implementation plan of the assigned matters. |  |
|  | PA4 | AI-enabled products and service can autonomously suggest what can be done. |  |
| **Self-Efficacy** | SE1 | I believe that I can use AI-enabled products and service even if there is no one around to tell me what to do as I go. | Delgosha et al., 2021; Li et al., 2021 |
|  | SE2 | I believe that I can use AI-enabled products and service if I have a lot of time to interact with. |  |
|  | SE3 | I believe that I can use AI-enabled products and service if I have the built-in help facility for assistance. |  |
| **Trust in AI** | TA1 | In general, I follow the advice given to me by AI-enabled products and service. | Chen et al., 2021; Li et al., 2021 |
|  | TA2 | I trust AI-enabled products and service performs can its duties well. |  |
|  | TA3 | I trust AI-enabled products and service will understand me better after constant communication and interaction. |  |
| **Community identification** | CI1 | I think my identity is similar to that of other community members. | Bagozzi et al., 2006 |
|  | CI2 | I feel that my identity is in line with the characteristics of this community. |  |
|  | CI3 | I really like being a member of this community. |  |
|  | CI4 | I feel that I have a strong emotional connection to this community. |  |
|  | CI5 | I consider myself an important member of the community. |  |
|  | CI6 | I feel I am a valuable member of the community. |  |
| **Customers engagement** | CE1 | I would like to know more about AI-enabled products and service. | Vivek et al., 2014 |
|  | CE2 | I track any information related to AI-enabled products and service. |  |
|  | CE3 | I pay close attention to all the information about AI-enabled products and service on the communities. |  |
|  | CE4 | Any information about AI-enabled products and service caught my attention. |  |
|  | CE5 | I spend a lot of my free time commenting or discussing about AI-enabled products and service on the communities. |  |
|  | CE6 | I am keen to participate in the related activities of AI-enabled products and service. |  |
|  | CE7 | I really enjoy using AI-enabled products and service. |  |
|  | CE8 | My life would be very different without AI-enabled products and service. |  |
|  | CE9 | I like my friends to participate in related activities of AI-enabled products and service with me. |  |
|  | CE10 | I enjoy using AI-enabled products and service when I participate with other people. |  |
|  | CE11 | I find it more interesting when people around me using AI-enabled products and service. |  |
| **Customer Participation Behavior** | CPB1 | Through the community, I can get information about AI-enabled products and service. | Lüthje, 2003; Yi & Gong, 2012 |
|  | CPB2 | I will actively respond to topics initiated by enterprises and seriously express my demands about AI-enabled products and service. |  |
|  | CPB3 | I will actively participate in AI-enabled products and service creativity collection activities and AI-enabled products and service performance evaluation activities carried out by the community. |  |
|  | CPB4 | I will participate in the promotion activities organized by communities about AI-enabled products and service. |  |
| **Customer Citizenship Behavior** | CCB1 | I often publish my own reviews about AI-enabled products and service to the community. | Koh, 2008; Tang et al., 2018 |
|  | CCB2 | I will actively promote AI-enabled products and service |  |
|  | CCB3 | I often participate in discussion of topics about AI-enabled products and service in the community and answer questions raised by other members. |  |
|  | CCB4 | I choose to forgive for AI-enabled products and service problems caused by a specific reason. |  |

**Table 3.**  Measurement Model

| **Construct** | **Item** | **Loadings** | **CR** | **AVE** | **Cronbach's Alpha** |
| --- | --- | --- | --- | --- | --- |
| Perceived Personalization | c1 | 0.7 | 0.843 | 0.519 | 0.843 |
|  | c2 | 0.74 |  |  |  |
|  | c3 | 0.757 |  |  |  |
|  | c4 | 0.686 |  |  |  |
|  | c5 | 0.715 |  |  |  |
| Perceived Autonomy | d1 | 0.72 | 0.824 | 0.539 | 0.822 |
|  | d2 | 0.745 |  |  |  |
|  | d3 | 0.774 |  |  |  |
|  | d4 | 0.695 |  |  |  |
| Community identification | f1 | 0.731 | 0.905 | 0.615 | 0.903 |
|  | f2 | 0.805 |  |  |  |
|  | f3 | 0.748 |  |  |  |
|  | f4 | 0.831 |  |  |  |
|  | f5 | 0.729 |  |  |  |
|  | f6 | 0.853 |  |  |  |
| Trust in AI | g1 | 0.752 | 0.807 | 0.583 | 0.805 |
|  | g2 | 0.766 |  |  |  |
|  | g3 | 0.772 |  |  |  |
| Self-Efficacy | h1 | 0.739 | 0.814 | 0.594 | 0.813 |
|  | h2 | 0.8 |  |  |  |
|  | h3 | 0.771 |  |  |  |
| Customers engagement | i1 | 0.806 | 0.872 | 0.532 | 0.87 |
|  | i2 | 0.688 |  |  |  |
|  | i3 | 0.702 |  |  |  |
|  | i4 | 0.76 |  |  |  |
|  | i5 | 0.706 |  |  |  |
|  | i6 | 0.709 |  |  |  |
| Customer Participation Behavior | j1 | 0.728 | 0.83 | 0.549 | 0.827 |
|  | j2 | 0.712 |  |  |  |
|  | j3 | 0.736 |  |  |  |
|  | j4 | 0.785 |  |  |  |
| Customer Citizenship Behavior | k1 | 0.865 | 0.919 | 0.738 | 0.887 |
|  | k2 | 0.854 |  |  |  |
|  | k3 | 0.845 |  |  |  |
|  | k4 | 0.872 |  |  |  |

**Table 4.** Discriminant Validity

| Construct | 1 | 2 | 3 | 4 | 5 | 6 | 7 | 8 |
| --- | --- | --- | --- | --- | --- | --- | --- | --- |
| Perceived Personalization | 0.72 |  |  |  |  |  |  |  |
| Perceived Autonomy | 0.38 | 0.734 |  |  |  |  |  |  |
| Social identification | 0.294 | 0.296 | 0.784 |  |  |  |  |  |
| Trust in AI | 0.352 | 0.295 | 0.236 | 0.764 |  |  |  |  |
| Self-Efficacy | 0.365 | 0.34 | 0.291 | 0.314 | 0.771 |  |  |  |
| Customers engagement | 0.396 | 0.393 | 0.362 | 0.39 | 0.405 | 0.729 |  |  |
| Customer Participation Behavior | 0.399 | 0.407 | 0.331 | 0.365 | 0.384 | 0.453 | 0.741 |  |
| Customer Citizenship Behavior | 0.383 | 0.396 | 0.384 | 0.355 | 0.387 | 0.489 | 0.363 | 0.859 |

**Table 5.** Hypothesis Testing (Direct Relationships)

| **Hypothesis** | **Relationship** | **Standardized Estimate** | **S.E.** | **C.R.** | **P** | **Decision** |
| --- | --- | --- | --- | --- | --- | --- |
| **H1a** | **PP 🡪 CPB** | 0.168 | 0.061 | 2.933 | 0.003 | Significant |
| **H2a** | **PA 🡪 CPB** | 0.225 | 0.058 | 3.986 | *** | Significant |
| **H3a** | **SE 🡪 CPB** | 0.183 | 0.051 | 3.365 | *** | Significant |
| **H4a** | **TA 🡪 CPB** | 0.196 | 0.055 | 3.476 | *** | Significant |
| **H5a** | **CI 🡪 CPB** | 0.117 | 0.045 | 2.491 | 0.013 | Significant |
| **H1b** | **PP** 🡪 **CCB** | 0.128 | 0.065 | 2.388 | 0.017 | Significant |
| **H2b** | **PA 🡪 CCB** | 0.188 | 0.061 | 3.591 | *** | Significant |
| **H3b** | **SE 🡪 CCB** | 0.164 | 0.054 | 3.225 | 0.001 | Significant |
| **H4b** | **TA 🡪 CCB** | 0.182 | 0.058 | 3.452 | *** | Significant |
| **H5b** | **CI 🡪 CCB** | 0.218 | 0.049 | 4.858 | *** | Significant |

PP= Perceived Personalization, PA= Perceived Autonomy, SE= Self-Efficacy, TA= Trust in AI, CI= Community identification, CPB= Customer Participation Behavior, CCB= Customer Citizenship Behavior.

**Table 6.** Indirect Relationships

| **Hypothesis** | **Path** | **Estimate** | **SE** | **LLCI** | **ULCI** | **p** | **Decision** |
| --- | --- | --- | --- | --- | --- | --- | --- |
| **H6a** | PP—CE—CPB | 0.027 | 0.016 | 0.004 | 0.072 | 0.013 | Significant |
| **H7a** | PA—CE—CPB | 0.032 | 0.017 | 0.008 | 0.078 | 0.006 | Significant |
| **H8a** | SE—CE—CPB | 0.04 | 0.018 | 0.013 | 0.087 | 0.002 | Significant |
| **H9a** | TA—CE—CPB | 0.039 | 0.019 | 0.009 | 0.087 | 0.004 | Significant |
| **H10a** | CI—CE—CPB | 0.031 | 0.016 | 0.007 | 0.072 | 0.004 | Significant |
| **H6b** | PP—CE—CCB | 0.037 | 0.018 | 0.007 | 0.081 | 0.013 | Significant |
| **H7b** | PA—CE—CCB | 0.044 | 0.019 | 0.014 | 0.09 | 0.005 | Significant |
| **H8b** | SE—CE—CCB | 0.055 | 0.019 | 0.023 | 0.101 | 0 | Significant |
| **H9b** | TA—CE—CCB | 0.053 | 0.02 | 0.02 | 0.101 | 0.001 | Significant |
| **H10b** | CI—CE—CCB | 0.042 | 0.017 | 0.015 | 0.086 | 0.002 | Significant |

PP= Perceived Personalization, PA= Perceived Autonomy, SE= Self-Efficacy, TA= Trust in AI, CI= Community identification, CPB= Customer Participation Behavior, CCB= Customer Citizenship Behavior.
